# Quantitative EEG parameters can improve the predictive value of the non-traumatic neurological ICU patient prognosis through the machine learning method

**DOI:** 10.3389/fneur.2022.897734

**Published:** 2022-07-28

**Authors:** Jia Tian, Yi Zhou, Hu Liu, Zhenzhen Qu, Limiao Zhang, Lidou Liu

**Affiliations:** ^1^Neurocritical Care Unit, Department of Neurology, The Second Hospital of Hebei Medical University, Shijiazhuang, China; ^2^Department of Neurology, The Second Hospital of Hebei Medical University, Shijiazhuang, China

**Keywords:** ICU, machine learning, quantitative electroencephalogram, prognosis, neurology

## Abstract

**Background:**

Better outcome prediction could assist in reliable classification of the illnesses in neurological intensive care unit (ICU) severity to support clinical decision-making. We developed a multifactorial model including quantitative electroencephalography (QEEG) parameters for outcome prediction of patients in neurological ICU.

**Methods:**

We retrospectively analyzed neurological ICU patients from November 2018 to November 2021. We used 3-month mortality as the outcome. Prediction models were created using a linear discriminant analysis (LDA) based on QEEG parameters, APACHEII score, and clinically relevant features. Additionally, we compared our best models with APACHEII score and Glasgow Coma Scale (GCS). The DeLong test was carried out to compare the ROC curves in different models.

**Results:**

A total of 110 patients were included and divided into a training set (n=80) and a validation set (*n* = 30). The best performing model had an AUC of 0.85 in the training set and an AUC of 0.82 in the validation set, which were better than that of GCS (training set 0.64, validation set 0.61). Models in which we selected only the 4 best QEEG parameters had an AUC of 0.77 in the training set and an AUC of 0.71 in the validation set, which were similar to that of APACHEII (training set 0.75, validation set 0.73). The models also identified the relative importance of each feature.

**Conclusion:**

Multifactorial machine learning models using QEEG parameters, clinical data, and APACHEII score have a better potential to predict 3-month mortality in non-traumatic patients in neurological ICU.

## Introduction

Compared with other critical illnesses, neurocritical illnesses have their characteristics, which are prominent neurological failures. Accurate prognostic indicators for neurological intensive care unit (ICU) patients are urgently needed to assist clinical management and counseling of patients and their families. As more and more patients benefited from intensive care technology and survived in the hospital, we are increasingly shifting our focus from in-hospital mortality to post-discharge outcomes.

At present, Glasgow Coma Scale (GCS) has been widely used for predicting the prognosis in the ICU for its simple, practical, and economic advantages (1). However, GCS does not reflect multiple organ damage in critically ill patients. Acute Physiology and Chronic Health Evaluation II (APACHEII) score (2), including the GCS score and the twelve physiological variables, theoretically include more considerations in predicting ICU patient outcomes. Studies have shown that other physiological variables excluding GCS in APACHE II are more accurate in predicting correct outcomes of late mortality (1).

Electroencephalography (EEG) is a non-invasive measure of cortical activity, especially suitable for sedation or comatose patients (3–5). Continuous EEG (cEEG) has been used as a part of routine multimodal monitoring in the neurological ICU to detect (non-convulsive or electrographic) seizures and ischemia (6). Quantitative EEG (QEEG), originated from raw EEG data, provides a quantitative analysis of the data both in the frequency and in the time domain. It transforms EEG elements into calculated parameters, simplifying the interpretation and allowing the analysis to be more objective (7, 8). In recent years, several studies have been proved that QEEG parameters could be used to detect the changes and predict prognosis in neurological ICU disease (9–13). However, more attention was paid on traumatic brain injury, the detection of epilepsy, and in-hospital mortality. Few studies have focused on non-traumatic neurocritical illnesses (which are also a major part of the neurology ICU) and post-discharge outcomes.

In this study, we used machine learning methods, combined with basic patient's characteristics, APACHEII score, and QEEG parameters, to create a new model. We hypothesize that a machine learning model for prognosis prediction would outperform traditional risk calculators, such as GCS and APACHEII.

## Methods

### Study population

We retrospectively analyzed neurological ICU patients from November 2018 to November 2021, The Second Hospital of Hebei Medical University. Inclusion criteria were as follows: (14) patients older than 18 years and (15) patients who were admitted to the neurological ICU. Exclusion criteria were as follows: (14) patients who were diagnosed with traumatic brain injuries; (15) patients who did not receive the EEG monitoring for some reasons (e.g., agitated patients or the local skull were absent after the operation or the EEG machine was a failure, etc.); (16) patient's pre-onset modified Rankin Scale (mRS) ≥ 2 points; (17) patients who had significant non-neurological diseases that seriously affect the prognosis, such as severe heart failure; (3) patients were conscious who got non-central nervous system diseases, such as Guillain–Barre syndrome and Myasthenia gravis. This retrospective study was approved by the Research Ethics Committee of the Second Hospital of Hebei Medical University (approval number: 2018-P031).

### Outcome assessment

We used 3-month mortality as the post-discharge outcome. It was performed by the telephone calls by 2 independently medical graduate students.

### Clinical parameters

We retrospectively collected the basic characters of patients (e.g., gender, age, and past medical history, etc.), GCS and APACHEII scores on admission, and several physiological variables which the worst at 24-h admission to ICU (the body temperature, mean arterial pressure, heart rate, serum sodium, serum potassium, and hematocrit) from the electronic medical records. We also collected the diagnoses. In the case where the patient had two, the “most severe” diagnoses would be considered.

### EEG recordings

Electroencephalography recordings were started as soon as possible after admission to the ICU. The patients were monitored using a digital video EEG monitor (Model Neusen. U) of Boruikang Technology (Changzhou) Co. Ltd, for at least 2 h each time, and at least once for each case. Ag/AgCl electrodes were placed according to the international 10–20 system. The impedance of each electrode was maintained below 5 kΩ, the sampling rate was 1,000 Hz, and the filtering range was 0.5–70 Hz. Data were stored in the European Data Format (EDF).

### QEEG parameters

The selected continuous EEG data with a length of about 30 min which from the first EEG monitoring data after admission without motion interference were analyzed quantitatively with the MATLAB R2015a (The MathWorks, Inc. MA, USA) software and its EEGLAB toolbox. The following steps were performed: (1) preprocessing: 50 Hz notch filtering was performed to remove power frequency interference; the third-order Butterworth filter was selected as the high-pass filter, the −3dB cutoff the frequency was 1 Hz; the 8th-order Butterworth filter was selected as the low-pass filter selects, and the −3dB cutoff frequency is 30 Hz; (2) adaptive noise reduction: eye electrical interference was removed using the method provided by the EEGLAB toolbox. All QEEG parameters were calculated for each 10-min window. For multiple epoch data, we adopt the method of extracting median, because it is found that median requires less data cleaning, which is also in line with common cognition.

#### Absolute power per band

The absolute power of the frequency bands was calculated by integration of the power spectral density (PSD) within each frequency band: delta (0.5–4 Hz), theta (4–8 Hz), alpha (8–13 Hz), and beta (13–20 Hz). Hereby, we estimated the PSD of each channel using Welch's method, with an overlap of 50%, and averaged the PSD over the 60 epochs within the 10-min windows.

#### Total power

The sum of all power bands (0.5–20 Hz) resulted in the total power.

#### Relative power per band

The relative power of each frequency band was defined as the ratio between the power within that frequency band and the total power.

*Alpha/delta ratio*The alpha/delta ratio (ADR) was calculated as the power ratio from the alpha (8–13 Hz) and delta (0.5–4 Hz) frequency bands.

#### Variability per frequency band

Variability in the power of each frequency band was computed by the ratio of the median absolute deviation (MAD) to the median power in each frequency band (18), resulting in a value between 0 (no variability) and 1 (high variability).

#### Brain symmetry index

The pairwise derived brain symmetry index (BSI) was used to calculate the symmetry of power between each pair of electrodes from the left and right hemisphere (19), expressed in a value between 0 (symmetric) and 1 (highly asymmetric). BSI was calculated over the frequency ranges 0.5–20 Hz (BSI ALL) and 0.5–4 Hz (BSI delta).

#### Mean amplitude

The mean amplitude (AMP) was defined as the standard deviation of the signal.

#### Regularity

Regularity (REG) is a measure for the continuity of the EEG pattern based on the variance of the amplitude of the signal. Regularity is normalized between 0 and 1, where a higher value indicates a signal with more regular amplitude (20).

### Multifactorial model

We chose the linear discriminant analysis (LDA), also known as Fisher linear discriminant (FLD). It is a classical algorithm of pattern recognition, which was introduced into the field of pattern recognition and artificial intelligence by Belhumeur in 1996 (21). The basic idea is to project the high-dimensional pattern samples into the optimal discriminant vector space, to extract classification information and compress the dimension of feature space. After the projection, the pattern samples have the maximum inter-class distance and the minimum intra-class distance in the new subspace. Therefore, it is an effective feature extraction method. It can guarantee the minimum intra-class distance and maximum inter-class distance of the projected pattern samples in the new space. That means the pattern has the best separability in this space.

First, we trained an LDA classifier that combined all QEEG parameters, APACHEII scores, and other characteristics, such as gender to predict patient mortality at 3 months. Second, some important features are selected and used to create a new model. In addition to the validation set, we also used 5-fold cross-validation to examine the performance of the machine learning classifiers. To evaluate model performance, we computed receiver operating characteristic (ROC) curves, the area under the curve (AUC), sensitivity, specificity, positive predictive value (PPV), and negative predictive value (NPV). The DeLong test was carried out to compare the ROC curves in different models.

### Statistical analysis

Statistical Package for the Social Sciences (SPSS) statistical software (version 26.0, SPSS Institute, Inc., Chicago, IL, USA) was used for data analysis. The measurement data for normal distribution were expressed as mean ± standard deviation and compared using the *t*-test. The skew distribution data were represented by median and quartile spacing, and a rank-sum test (Mann–Whitney U test) was used for comparison between groups. Counting data were represented by frequency and compared using the chi-square test. The significance level was set at *p* < 0.05.

## Results

### Patient demographics

**Figure 1:**
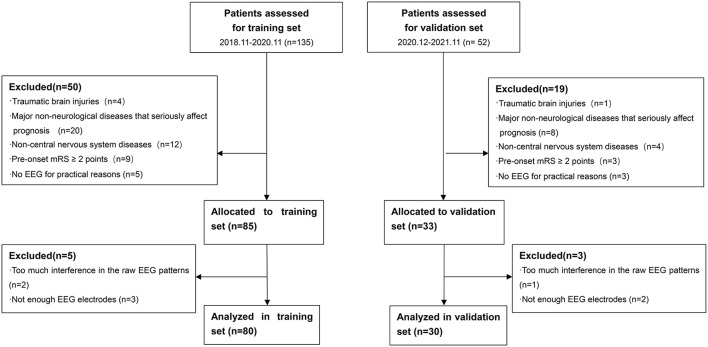
Flow diagram for inclusion and exclusion of eligible patients. mRS, modified Rankin Scale; EEG, Electroencephalography.

There were totally 110 patients included in our study. A total of 80 patients from November 2018 to November 2020 were enrolled in the training set. A total of 30 patients from December 2020 to November 2021 were enrolled in the validation set. A flow diagram for the inclusion and exclusion of eligible patients is shown in Figure 1. Table 1 shows the baseline characteristics of both training and validation sets. In training set, there were 53 (66.25%) patients in the survival group and 27 (33.75%) patients in the non-survival group. Patients in the survival group had lower APACHEII scores (*p* < 0.001) and higher GCS scores (*p* = 0.015). More patients in the non-survival group had a previous medical history of diabetes (*p* < 0.001) and the administration of norepinephrine (*p* = 0.003). The age, gender, the diagnoses, the medication of analgesic and sedative drugs, the ICU stay, and the EEG start in hours after onset did not differ between the groups.

**Table 1:** Baseline characteristics of training and validation sets.

**Characteristic**	**Training set**			***p*** **-value**	**Validation set**			***p*** **-value**
	**Total** **(*n* = 80)**	**Survival** **group** **(*n* = 53)**	**Non-survival group** **(*n* = 27)**		**Total** **(*n* = 30)**	**Survival** **group** **(*n* = 20)**	**Non-survival group** **(*n* = 10)**	
Age, year, Mean ± SD	58.0 (38.5, 68.8)	58.0 (31.0, 67.5)	59.8 ± 13.4	0.107	50.5 ± 20.6	49.4 ± 21.3	51.6 ± 20.7	0.834
Male, *n* (%)	46 (57.5%)	37 (69.8%)	9 (33.3%)	0.774	18 (60.0%)	10 (50.0%)	8 (80.0%)	0.114
Admission APACHE II, median (IQR)	17.0 (15.0, 20.8)	16.0 (13.5, 19.0)	20.0 (17.0, 25.0)	<0.001	17.0 (15.0, 19.0)	16.5 (15.0, 18.0)	20.7 ± 5.4	0.034
Admission GCS, median (IQR)	4.0 (3.0, 6.0)	5.0 (3.0, 7.5)	3.0 (3.0, 5.0)	0.015	6.0 (4.0, 8.0)	6.0 (5.0, 8.8)	5.5 ± 2.0	0.338
Previous medical history, *n* (%)								
Hypertension	42 (52.5%)	25 (47.2%)	17 (63.0%)	0.181	15 (50%)	9 (45.0%)	6 (20.0%)	0.439
CHD	17 (21.3%)	9 (17.0%)	8 (29.6%)	0.191	7 (23.3%)	5 (25.0%)	2 (20.0%)	0.760
Diabetes	11 (13.8%)	2 (3.8%)	9 (33.3%)	<0.001	6 (20.0%)	4 (20.0%)	2 (20.0%)	1.000
Diagnose, *n* (%)								
Hypoxic ischemic encephalopathy	6 (7.5%)	5 (9.4%)	1 (3.7%)	0.658	3 (8.8%)	2 (10.0%)	1 (10.0%)	1.000
Intracerebral hemorrhage	9 (11.3%)	6 (11.3%)	3 (11.1%)	1.000	2 (5.9%)	1 (5.0%)	1 (10.0%)	0.605
Cerebral ischemic stroke	36 (45.0%)	20 (37.7%)	16 (59.3%)	0.067	6 (17.6%)	4 (20.0%)	2 (20.0%)	1.000
Central nervous system infectious diseases	25 (31.3%)	19 (35.8%)	6 (22.2%)	0.214	11 (32.4%)	7 (35.0%)	4 (40.0%)	0.789
Other diseases/Unknown	4 (5.0%)	3 (5.7%)	1 (3.7%)	1.000	8 (23.5%)	6 (30.0%)	2 (20.0%)	0.559
Medication administration, *n* (%)								
Propofol	12 (15.0%)	9 (20.8%)	3 (11.1%)	0.487	6 (20.0%)	3 (15.0%)	3 (30.0%)	0.333
Midazolam	60 (75.0%)	40 (75.5%)	20 (74.1%)	0.891	28 (93.3%)	18 (90.0%)	10 (100.0%)	0.301
Fentanyl	49 (61.3%)	31 (58.5%)	18 (66.7%)	0.478	24 (80.0%)	15 (75.0%)	9 (90.0%)	0.333
Noradrenaline	11 (13.8%)	3 (5.7%)	8 (29.6%)	0.003	3 (10.0%)	1 (5.0%)	2 (20.0%)	0.197
Length of ICU say, d	23.5 (15.0, 40.0)	20.0 (15.0, 29.0)	29.0 (15.0, 52.5)	0.163	26 (17.0, 28.0)	25.5 (17.0, 27.5)	24.4 ± 6.5	0.522
EEG start in hours after onset (median (IQR))	3.5 (1.75, 6.25)	3.0 (1.00, 6.00)	4.0 (2.00, 10.50)	0.141	4 (2.0, 10.0)	4.0 (2.00, 11.00)	3.0 (1.50, 6.50)	0.472

*APACHEII, Acute Physiology and Chronic Health Evaluation II; GCS, Glasgow Coma Scale; CHD, coronary heart disease; ICU, intensive care unit; IQR, interquartile range; SD, standard deviation. The bold values mean P <0.05*.

In validation set, there were 20 (66.67%) patients in the survival group and 10 (33.33%) patients in the non-survival group. Patients in the survival group also had lower APACHEII scores (*p* = 0.034). There were no significant differences in other characteristics between the groups.

### Model selection and performance in the training set

**Figure 2:**
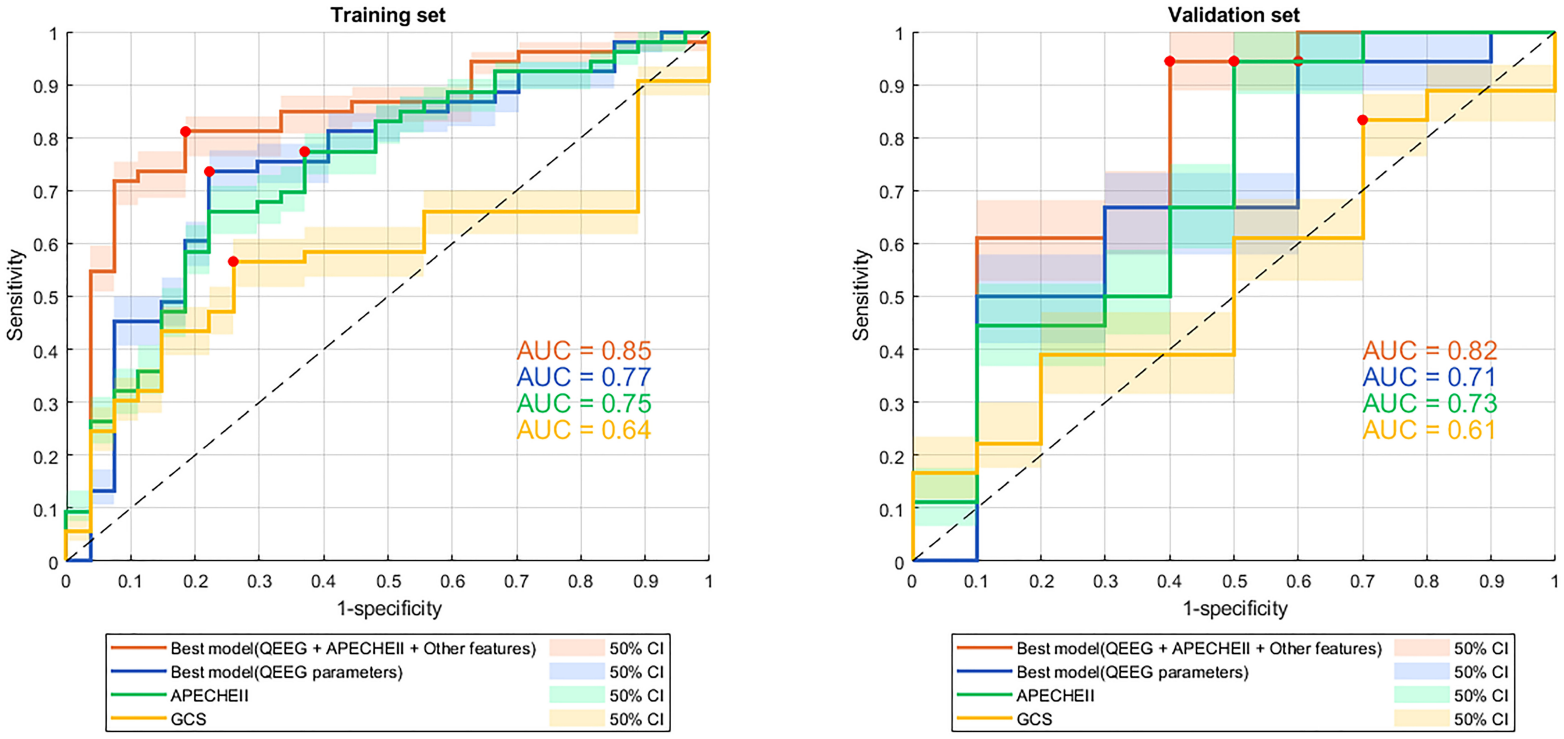
ROC curves with 50% confidence interval of models and scores for predicting 3-month mortality. ROC curve, receiver operating characteristic curve; QEEG, quantitative EEG; APACHEII, Acute Physiology and Chronic Health Evaluation II; GCS, Glasgow Coma Scale; AUC, area under the curve. The red dots indicate the threshold at which the sensitivity and specificity are best.

Our best model based on all QEEG parameters, APACHEII, and other features had an AUC of 0.85 (specificity 0.81, sensitivity 0.81), which was the highest AUC value among other model and scores. Since GCS and age are included in APACHEII's calculation formula, only APACHEII features were added when training the best model. Models in which we selected only the 4 best QEEG parameters (delta power rate, beta power rate, theta power rate, and alpha power rate) had an AUC of 0.77, which was similar to that of APACHEII (AUC=0.75), and much higher than that of GCS (AUC = 0.64). The results of the DeLong test indicated that the AUC of the best models was significantly better than that of GCS (Table 2, Figure 2).

**Table 2:** The diagnosis results of the scores and the models.

	**Variables**	**AUC**	**Threshold**	**Sensitivity**	**Specificity**	**PPV**	**NPV**
Training set (50%CI)	GCS	0.64 (0.60–0.68)	10	0.57 (0.52–0.61)	0.74 (0.68–0.80)	0.81 (0.79–0.84)	0.47 (0.46–0.47)
	APACHEII	0.75 (0.71–0.79)	19	0.77 (0.72–0.81)	0.63 (0.57–0.69)	0.80 (0.78–0.82)	0.59 (0.56–0.60)
	Best model (QEEG parameters)	0.77 (0.73–0.80)*	0.64	0.74 (0.70–0.78)	0.78 (0.71–0.83)	0.87 (0.84–0.88)	0.60 (0.58–0.63)
	Best model (QEEG+APACHEII+other features)	0.85 (0.81–0.87)*	0.60	0.81 (0.78–0.85)	0.81 (0.75–0.86)	0.91 (0.90–0.97)	0.69 (0.66–0.72)
Validation set (50%CI)	GCS	0.61 (0.53–0.68)	10	0.83 (0.75–0.88)	0.30 (0.22–0.43)	0.68 (0.67–0.70)	0.50 (0.49–0.51)
	APACHEII	0.73 (0.63–0.79)	18	0.94 (0.89–1.00)	0.50 (0.38–0.60)	0.77 (0.74–0.80)	0.83 (0.75–1.00)
	Best model (QEEG parameters)	0.71 (0.63–0.77)	0.68	0.94 (0.89–0.96)	0.40 (0.30–0.50)	0.74 (0.71–0.76)	0.79 (0.71–0.80)
	Best model (QEEG+APACHEII+other features)	0.82 (0.74–0.86)^*†^	0.72	0.94 (0.89–1.00)	0.60 (0.50–0.70)	0.81 (0.78–0.84)	0.86 (0.78–1.00)

*AUC, area under the curve; PPV, positive predictive value; NPV, negative predictive value; GCS, Glasgow Coma Scale; APACHEII, Acute Physiology and Chronic Health Evaluation II; QEEG parameters, delta power rate, beta power rate, theta power rate, and alpha power rate.*

*DeLong test indicated that there were statistical differences in the AUC from GCS.

*DeLong test indicated that there were statistical differences in the AUC from best model (QEEG parameters)*.

### Performance in the validation set

Our best model classified 3-month mortality in the validation set had an AUC of 0.82 which based on all QEEG parameters, APACHEII, and other features. It was also the highest AUC value among other models and scores. Model in which we selected only the 4 best QEEG parameters had an AUC of 0.71, which was similar to that of APACHEII. The AUC of the APACHEII to classified 3-month mortality in the validation set is 0.73. Both model with 4 QEEG parameters and APACHEII had the same sensitivity of 0.94. GCS also had the lowest AUC for classified 3-month mortality (AUC = 0.61). The results of the DeLong test indicated that the AUC of the best model, which based on all QEEG parameters, APACHEII, and other features, was significantly better than that of GCS (Table 2, Figure 2).

### Feature contributions

**Figure 3:**
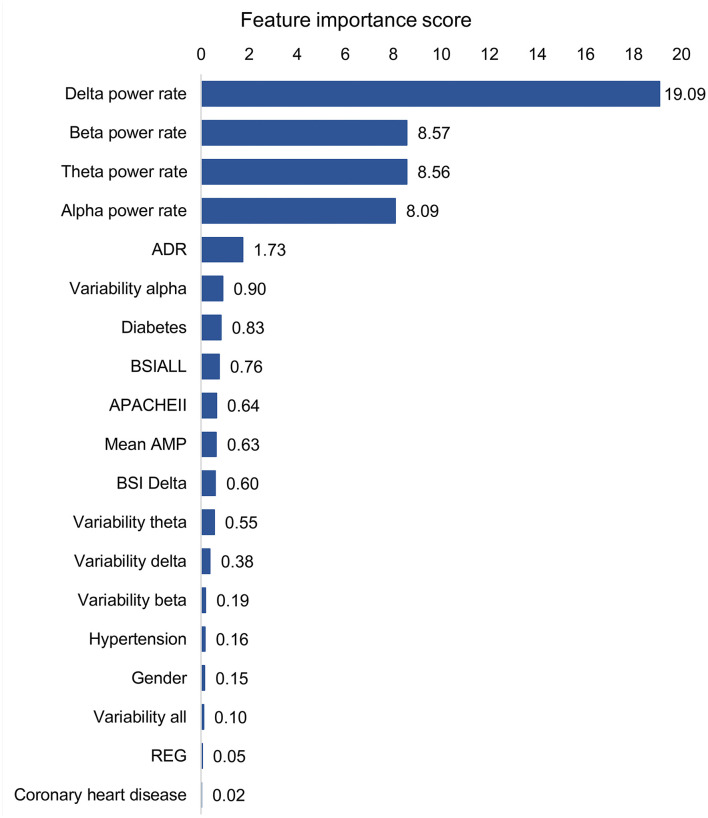
Feature contribution of the best model based on all QEEG parameters, APACHEII, and other features. ADR, alpha/delta ratio; BSI, brain symmetry index; APACHEII, Acute Physiology and Chronic Health Evaluation II; Mean AMP, mean amplitude; REG, regularity.

**Figure 4:**
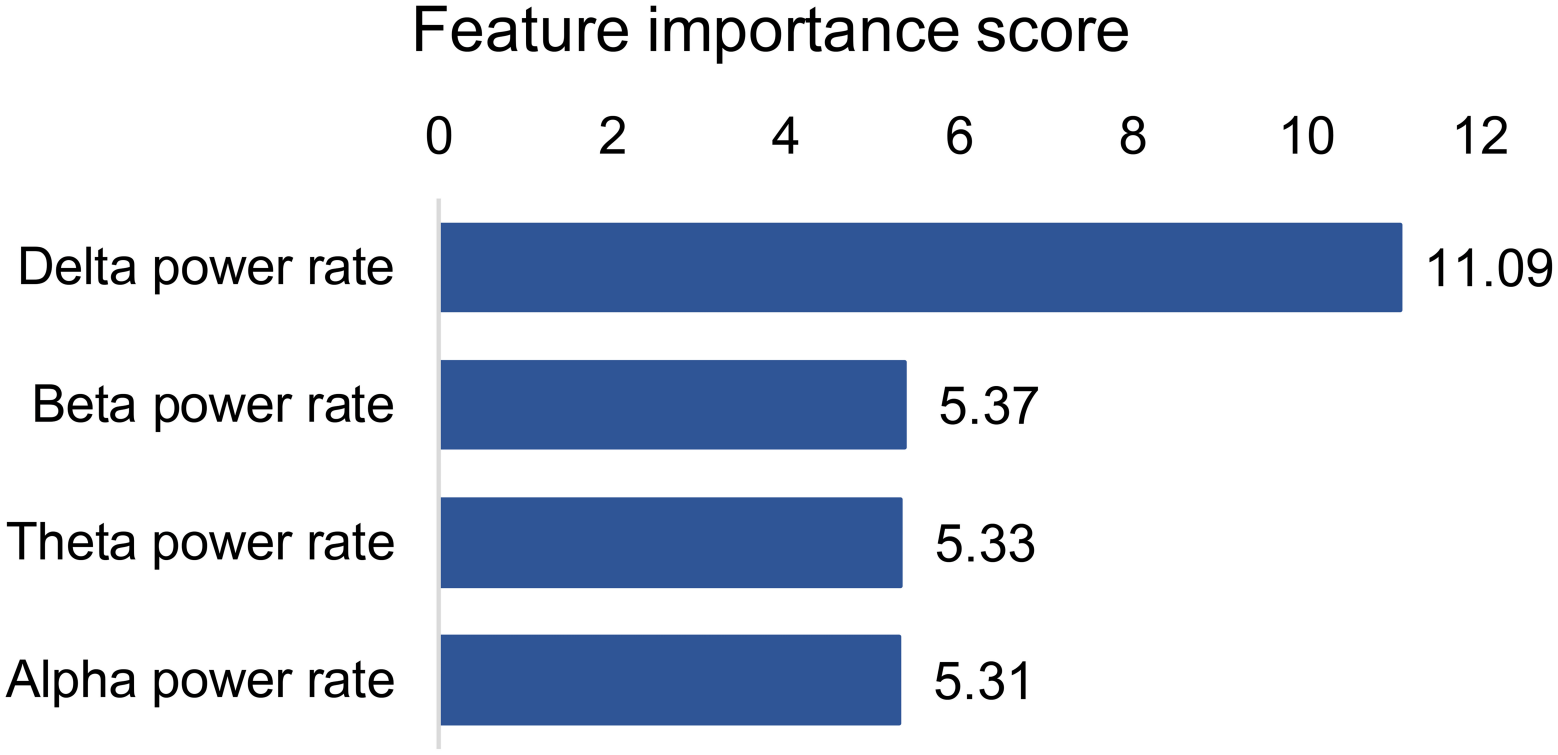
Feature contribution of the best model based on four QEEG parameters.

The best models used 19 features (14 QEEG parameters, past medical histories, APACHEII, and gender), of which each contribution is shown in Figure 3. The 4 QEEG parameters (delta power rate, beta power rate, theta power rate, and alpha power rate) were more important than other features. Specifically, the delta power rate had the highest feature importance score of 19.09. However, other features had much lower feature importance scores. The least relevant was the previous history of coronary heart disease and hypertension, 2 QEEG parameters (REG and variability all), and gender, the feature importance score of which were all <0.20. The APACHEII score is also lower than the part of QEEG parameters (delta power rate, beta power rate, theta power rate, alpha power rate, ADR, variability alpha, and BSIALL). The best model used only 4 QEEG parameters (delta power rate, beta power rate, theta power rate, and alpha power rate), of which each contribution is shown in Figure 4. The delta power rate also got the highest feature importance score of 11.09. The other 3 QEEG parameters got similar feature importance scores.

## Discussion

More reliable and accurate individual predictions of mortality through better models may improve clinical decision-making, leading to better risk-benefit assessments and improved individual management.

Several studies have attempted to correlate individual QEEG parameters with patient outcomes in a variety of non-traumatic neurological disorders (9, 17, 22, 23), and a few studies have shown that models based on multiple QEEG parameters and machine learning methods can predict the post-discharge outcomes of neurological ICU patients who always got non-traumatic neurocritical illnesses. We developed a model using a machine learning method that included multiple QEEG parameters, APACHEII scores, and patient general baseline data. With both specificity and sensitivity of 0.81, the DeLong test indicated that it had a higher diagnostic value than GCS for 3-month mortality in neurological ICU patients (Table 2, Figure 2). Although the AUCs between the best model based on all QEEG parameters, APACHEII, and other features and APACHEII did not reach a statistical difference, we could see a better trend toward from the machine learning model. At the meantime, we found that in the contribution score of this model, the contribution of QEEG parameters was the most important, which exceeds that of APACHEII score. It can also be seen from Table 2 that the diagnostic value of the model containing only 4 QEEG parameters is similar to that of APACHEII in both training and validation sets (AUC of 0.77 vs. 0.75 in the training set and 0.71 vs. 0.73 in the validation set). These data suggest that multifactorial models may benefit from including QEEG parameters. So, we want to emphasize that machine learning models that include QEEG parameters may have better predictive value than traditional models. Our results support this possibility, and the machine learning model can be further optimized in the future to improve this possibility.

Delta power rate contributes most to the prediction models, which has been confirmed in many studies that delta power rate was a correlation with poor outcomes (24, 25). Interestingly, the predictive contribution of the 4 basic band power rates was the most important compared to other QEEG parameters. This is not consistent with the traumatic brain injury (TBI) findings (11, 18). In patients with TBI, mean AMP, alpha power, and variability of the relative fast theta power were found to contribute significantly among QEEG parameters. In terms of contribution score, in addition to the 4 basic band power rates, ADR, variability alpha, and BSIALL rank ahead of APACHEII score. The possible reason is that the population in this study includes multiple diseases, and the prognosis of cerebral hemorrhage, cerebral infarction, and hypoxic-ischemic encephalopathy (HIE) is correlated with the power of the 4 basic band power rates. But so far, there are few studies on the prognosis of central nervous system infection using EEG, and more attention is paid to infection indicators. ADR and variability alpha are associated with cerebral ischemia, especially for the recognition of delayed cerebral ischemia after subarachnoid hemorrhage (26–30). BSI is a good predictor of stroke prognosis (31, 32). The reason may be the cerebrovascular patients account for more patients in the training set, so BSIALL has a higher contribution. Patients with HIE are usually the cases with symmetrical and diffuse brain lesions. So theoretically, BSI should not be prognostic for HIE. It is worth mentioning that in the validation set, cerebrovascular diseases do not occupy the largest proportion of diseases, but instead central nervous system infection. Even so, our training models also obtained good verification results, indicating that our models were generally applicable to the prognosis of patients in neurological ICU.

Cho et al. (1) found that for prediction of the late mortality, the APACHE III and II had better accuracy than GCS. Our results also showed that APACHEII score might have a higher diagnostic value than the GCS score, but the result did not reach the statistical difference. APACHEII score had a higher predictive ability of post-discharge outcome than GCS, which might be due to the addition of more physiological parameters. However, between the two models established by our machine learning method, the predictive value of only 4 QEEG parameters was similar to that of APACHEII score and higher than that of GCS score, indicating that the diagnostic value can also be improved by QEEG parameters from the perspective of brain function. In other words, QEEG parameters may be more valuable than GCS in evaluating brain function. Additionally, it suggests that QEEG parameters alone may be a promising alternative to APACHEII score and GCS score in neurological ICU prognosis prediction for the discharge outcome, which requires the validation of larger data and depends on the popularity of QEEG technology in ICU.

Sedative drugs can affect EEG presentation; however, the effect is likely to reduce the contribution of QEEG parameters (16). In both training and validation sets, there were no statistically significant differences in the use of sedatives between the two groups. So, sedative drugs may not have a significant effect on the results. Besides, studies that exclude sedatives from ICU patients are sometimes unethical. We focused on quantitative analysis instead of visual EEG analysis, so epilepsy was not included in the analysis. Quantitative EEG processing is still carried out to follow the methods, and the epileptic part is not artificially excluded.

Since the endpoint was the outcome after discharge, the specific cause of death could not be counted for many patients, but it was likely that the part of which was caused by non-nervous system organ failure. So, the optimal model including multiple parameters had the highest diagnostic value. In addition to APACHEII and GCS, there is also a difference between the two groups in the history of diabetes in training set (Table 1). Additionally, the contribution score of diabetes history was also slightly higher than that of APCAHEII (Figure 3). So, the history of diabetes may influence the prognosis. This may be due to diabetes in ICU patients is associated with non-nervous system organ dysfunction, such as infection (15) and acute kidney injury (33).

We also run our models on the patients who included pre-onset mRS ≥ 2. The results showed that the AUC of GCS increased, and there was no statistical difference of AUCs among models and GCS in the training set (Supplementary Figure S1, Supplementary Table S1). It may be due to the patients, who pre-onset mRS <2, at the early onset did not reach a severe GCS. Additionally, patients with pre-onset mRS ≥ 2 points may have severe GCS at an early stage, so the prognostic value is higher. These results should be interpreted with caution because the patients with pre-onset mRS ≥ 2 were few. Our study more focused on the population with new brain injury, not on the population with disability from previous brain injury. Future studies on this population can be carried out independently for more reliable results.

While this study showed promising results, there are several limitations. First, this study had a relatively small sample size, while we used cross-validation to overcome the limitations of the small sample size in training set. From the results of the validation set, our models were also well-verified. In this proof-of-concept study, we demonstrate the potential of machine learning models in this regard. These results should be validated in a larger study. Second, the subjects of our study were patients in neurological ICU, including several common diseases, without targeting a specific disease. However, from the perspective of single-factor analysis, disease diagnosis does not constitute a risk factor for outcome (Table 1). Since the proportions of diseases in the training set and the validation set are different (cerebrovascular diseases in the training set account for the largest proportion, and central nervous system infectious diseases in the validation set account for the largest proportion), our results are not specific to the diagnosis of any disease, but generally applicable to most of the neurocritical illnesses in neurological ICU. We believe that EEG ultimately reflects the level of bioelectrical activity of cortical cells and can reflect the severity of any disease in which brain function is impaired. Of course, QEEG can be explained in specific diseases, and different neurocritical illnesses may have different QEEG parameters to respond to different needs. We also need QEEG parameters for comparing severity among different illnesses. Müller et al. (34) found that visual EEG can predict the prognosis of coma patients with different causes. Third, HIE accounts for a small proportion in both training and validation sets. Since we focused on quantitative analysis instead of visual EEG analysis, some EEG characteristics (background continuous and background reactivity) were not incorporated in our proof-of-concept study, which are more important for HIE (14, 34). These deficiencies may reduce the value of our models in evaluating the prognosis of HIE groups. However, our research aims to establish a model that is generally applicable to multiple diseases, rather than a specific disease. Fourth, we did not select many clinical indicators (many of the clinical indicators originally selected were removed by dimensionality reduction in the classifier during modeling), which may exaggerate the contribution of QEEG parameters. However, our results have reflected the advantages of machine learning methods and QEEG parameters compared with traditional scores. Fifth, we chose only a single machine learning approach, to offer the possibility. Multiple combinations of machine learning might improve diagnostic value.

## Conclusion

Multifactorial machine learning model using QEEG parameters, clinical data, and APACHEII score have a better potential to predict 3-month mortality in non-traumatic patients in neurological ICU.

## Data availability statement

The raw data supporting the conclusions of this article will be made available by the authors, without undue reservation.

## Ethics statement

The studies involving human participants were reviewed and approved by the Ethics Committees of the Second Hospital of Hebei Medical University. The patients/participants provided their written informed consent to participate in this study. Written informed consent was obtained from the individual(s) for the publication of any potentially identifiable images or data included in this article.

## Author contributions

JT and LL conceived and designed the study, and wrote the article. YZ and HL performed data acquisition. ZQ and LZ analyzed the data. All authors read and approved the final manuscript. All authors contributed to the article and approved the submitted version.

## Conflict of interest

The authors declare that the research was conducted in the absence of any commercial or financial relationships that could be construed as a potential conflict of interest.

## Publisher's note

All claims expressed in this article are solely those of the authors and do not necessarily represent those of their affiliated organizations, or those of the publisher, the editors and the reviewers. Any product that may be evaluated in this article, or claim that may be made by its manufacturer, is not guaranteed or endorsed by the publisher.

## Supplementary material

Supplementary Figure S1 ROC curves with 50% confidence interval of models and scores for predicting 3-month mortality with patients included pre-onset mRS ≥ 2. ROC curve, receiver operating characteristic curve; mRS, modified Rankin Scale; QEEG, quantitative EEG; APACHEII, Acute Physiology and Chronic Health Evaluation II; GCS, Glasgow Coma Scale; AUC, area under the curve. The red dots indicate the threshold at which the sensitivity and specificity are best.

Supplementary Table S1 The diagnosis results of the scores and the models (patients include mRS ≥ 2).

## Abbreviations

ICU: intensive care unit
QEEG: quantitative electroencephalography
APACHEII: Acute Physiology and Chronic Health Evaluation II
LDA: linear discriminant analysis
GCS: Glasgow Coma Scale
cEEG: continuous EEG
mRS: modified Rankin Scale
PSD: power spectral density
ADR: alpha/delta ratio
MAD: median absolute deviation
BSI: brain symmetry index
AMP: mean amplitude
REG: regularity
FLD: Fisher linear discriminant
ROC: receiver operating characteristic
AUC: area under the curve
PPV: positive predictive value
NPV: negative predictive value.
