# Supplementary figures and images for: Quantitative EEG parameters can improve the predictive value of the non-traumatic neurological ICU patient prognosis through the machine learning method
Source: Front Neurol. 2022 Jul 28;13:897734. doi: 10.3389/fneur.2022.897734 (PMC9366714; doi:10.3389/fneur.2022.897734)

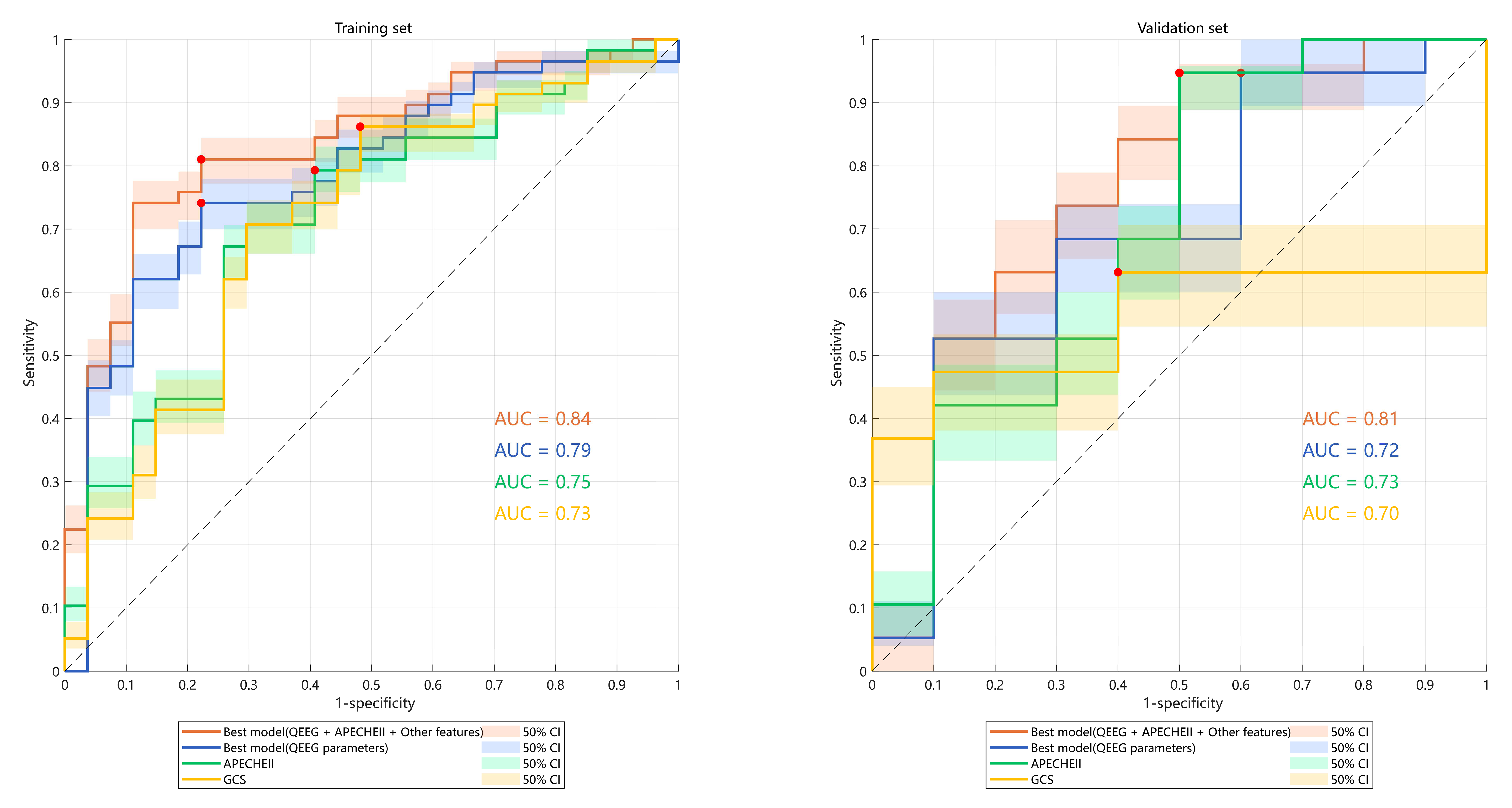

Supplement: Supplementary Figure S1 — ROC curves with 50% confidence interval of models and scores for predicting 3-month mortality with patients included pre-onset mRS ≥ 2. ROC curve, receiver operating characteristic curve; mRS, modified Rankin Scale; QEEG, quantitative EEG; APACHEII, Acute Physiology and Chronic Health Evaluation II; GCS, Glasgow Coma Scale; AUC, area under the curve. The red dots indicate the threshold at which the sensitivity and specificity are best. [file Image_1.TIF]
