# Supplementary material for: Quantitative EEG parameters can improve the predictive value of the non-traumatic neurological ICU patient prognosis through the machine learning method
Source: Front Neurol. 2022 Jul 28;13:897734. doi: 10.3389/fneur.2022.897734 (PMC9366714; doi:10.3389/fneur.2022.897734)
Supplement: Supplementary Table S1 — The diagnosis results of the scores and the models (patients include mRS ≥ 2). [file Table_1.DOCX]

**Supplementary Table 1.** The diagnosis results of the scores and the models. (patients include mRS ≥ 2)

| **Variables** | | **AUC** | **threshold** | **Sensitivity** | **Specificity** | **PPV** | **NPV** |
| --- | --- | --- | --- | --- | --- | --- | --- |
| Training set(50%CI) | GCS | 0.73(0.69-0.76) | 10 | 0.86(0.83-0.89) | 0.52(0.45-0.59) | 0.79(0.78-0.81) | 0.64(0.61-0.66) |
|  | APACHEII | 0.75(0.71-0.79) | 19 | 0.79(0.76-0.83) | 0.59(0.52-0.65) | 0.81(0.79-0.83) | 0.57(0.56-0.59) |
|  | Best model (QEEG parameters) | 0.79(0.76-0.87) | 0.68 | 0.74(0.70-0.78) | 0.78(0.71-0.83) | 0.88(0.85-0.90) | 0.58(0.56-0.60) |
|  | Best model (QEEG+APACHEII+other features) | 0.84(0.80-0.87) | 0.62 | 0.81(0.77-0.84) | 0.81(0.75-0.86) | 0.89(0.86-0.90) | 0.66(0.62-0.68) |
| Validation set(50%CI) | GCS | 0.70(0.63-0.75) | 10 | 0.63(0.55-0.70) | 0.60(0.50-0.70) | 0.75(0.73-0.78) | 0.46(0.45-0.47) |
|  | APACHEII | 0.73(0.65-0.79)* | 18 | 0.95(0.89-0.96) | 0.50(0.38-0.60) | 0.78(0.74-0.80) | 0.82(0.74-0.83) |
|  | Best model (QEEG parameters) | 0.72(0.65-0.79) | 0.68 | 0.95(0.90-1.00) | 0.40(0.29-0.50) | 0.75(0.73-0.77) | 0.80(0.72-1.00) |
|  | Best model (QEEG+APACHEII+other features) | 0.81(0.73-0.86)*† | 0.72 | 0.95(0.89-0.96) | 0.50(0.40-0.62) | 0.78(0.75-0.81) | 0.82(0.74-0.83) |

AUC: area under the curve; PPV: positive predictive value; NPV: negative predictive value; GCS: Glasgow Coma Scale; APACHEII: cute Physiology and Chronic Health Evaluation II; QEEG parameters: delta power rate, beta power rate, theta power rate, and alpha power rate.

*DeLong test indicated that there were statistical differences in the AUCs with GCS.

†DeLong test indicated that there were statistical differences in the AUCs with Best model (QEEG parameters).
